# Factors influencing efficacy of neural stem and progenitor cell transplantation

**DOI:** 10.4103/NRR.NRR-D-25-01140

**Published:** 2026-01-27

**Authors:** Angelina Baltazar, Jennifer N. Dulin

**Affiliations:** Department of Neuroscience, Perelman School of Medicine, University of Pennsylvania, Philadelphia, PA, USA; Department of Biology, Texas A&M University, College Station, TX, USA; Texas A&M Institute for Neuroscience, Texas A&M University, College Station, TX, USA

Neural stem and progenitor cell (NSPC) transplantation holds promise for treating neurological injuries and diseases, yet the mechanisms underlying therapeutic efficacy remain incompletely understood. This Perspective highlights emerging evidence that synaptic connectivity between transplanted cells and host circuits is a key determinant of functional recovery. Drawing on analyses in spinal cord injury, traumatic brain injury, and Parkinson’s disease (PD), we argue that factors such as donor cell identity, graft-host compatibility, and neuronal phenotype critically influence outcomes. A deeper understanding of circuit integration will be essential for optimizing NSPC-based therapies and advancing clinical translation.

NSPC transplantation has emerged as a promising treatment option for replacing lost neuronal populations and reconstructing neural circuits in the central nervous system following injury or disease. While a great deal of knowledge has come from NSPC grafting experiments conducted in small and large animal models, there has been advancement towards clinical translation of cell therapies for multiple neurological conditions. However, there remain many fundamental questions regarding the therapeutic mechanisms of neural cell replacement therapies that must be answered before clinical efficacy can be achieved.

**Figure 1:**
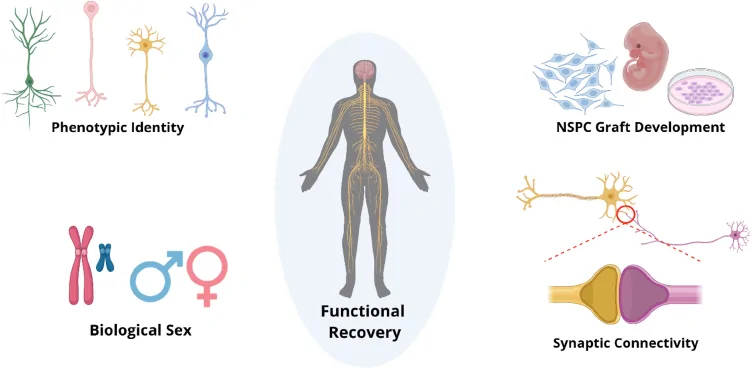
Factors contributing to functional recovery post spinal cord injury and other neurological injuries and diseases. Factors included but not limited to the biological sex of the donor neural stem and progenitor cells (NSPCs) (bottom left), phenotypic identity of the NSPC transplant (top left), NSPC graft development with age (top right), and synaptic connectivity between the host injured environment and NSPC transplant neuronal circuits should be considered when designing NSPC transplantation therapeutics for spinal cord injury and other neurological injuries and diseases. Created with BioRender.com.

Over the past decade, progress has unfolded in distinct stage, from early proof-of-concept evidence showing that NSPCs can form functional relays, to refinement of graft specificity, and more recently to engineered and phenotype-directed approaches. In this Perspectives article, we will argue that synaptic connectivity is needed for functional recovery after neurological disease and/or trauma. We will discuss evidence utilizing diverse disease and injury models, such as spinal cord injury (SCI), traumatic brain injury, and PD. We will show that NSPC transplantation analyses that achieve synaptic integration between host and graft have a higher potential for improved recovery, highlighting how factors influencing efficacy, such as donor identity, host/graft compatibility, neuronal phenotype, and engineering strategies, have emerged over time to shape the therapeutic potential of NSPCs (**Figure 1**).

**Early proof-of-concept studies in neural stem and progenitor cell transplantation:** The first study to demonstrate synaptic connectivity through NSPC grafts was authored by the Fischer lab (Bonner et al., 2011). In the paper, the authors transplanted a mixed population of neuronal and glial-restricted precursors from the embryonic rat spinal cord into a C1 SCI in rats. One week later, they injected brain-derived neurotrophic factor-expressing lentivirus into the dorsal column neurons to guide the axons to the intended target. Their goal was to form neuronal relays between the injured dorsal column sensory axons and the denervated dorsal column nuclei. After 6 weeks, they observed excitatory synaptic connections between the host circuitry and grafted neurons via immunoelectron microscopy. In addition, synaptic activity was observed via stimulus-evoked c-Fos expression and electrophysiological recordings. Together, their results demonstrate that transplanted NSPCs can form neuronal relays with the host circuitry by extending across the SCI site, establishing a critical step to consider for neuronal repair (Bonner et al., 2011). This paper provided the first clear demonstration that transplanted NSPCs could form neuronal relays with host circuitry, laying a foundation for subsequent work.

In parallel, work in the adult mouse cortex showed that area-specific identity was critical for graft success. Michelsen et al. (2015) transplanted cortical neurons derived from embryonic stem cells into the lesioned visual cortex and found that only in this matched environment did grafts extend axons and integrate electrophysiologically. The authors differentiated mouse embryonic stem cells into neurons of visual cortex identity, which demonstrates the potential to restore damaged pathways and synaptic connections of the adult mouse visual cortex through long-range axon projections between the injured and host circuitry and electrophysiological recordings. When the same neurons were transplanted into the motor cortex, these effects were not observed. This indicated that integration requires a match in the areal identity of grafted and lesioned neurons (Michelsen et al., 2015). This study underscored the importance of regional identity in graft/host connectivity following transplantation into the injured nervous system.

**Regional specificity and connectivity of neural stem and progenitor cell grafts:** Kikuchi et al. (2017) demonstrated long-term survival and function of human induced pluripotent stem cell-derived dopaminergic grafts in primate models of PD, with robust neurite extension and behavioral recovery. They found that NPSC graft-derived dopaminergic neurons had extended dense neurites into the host striatum, and did not lead to the formation of tumors. This resulted in a reduced immune response and an increase in spontaneous movement in the primate models, which was tested using magnetic resonance imaging, positron emission tomography, and video-based analysis (Kikuchi et al., 2017). Espuny-Camacho et al. (2018) extended the concept of cortical specificity by showing that human embryonic stem cell-derived cortical neurons integrated into lesioned adult mouse cortex in an area-specific manner, projecting axons appropriately and forming synaptic connections. The graft composed of neurons that represented six cortical layers, projected axons to specific cortical targets in the host circuitry and were synaptically active in the host environment. While transplanting these neurons into the visual cortex shows specific restoration of damaged cortical pathways, these results were not seen when transplanting the same neurons into the motor cortex (Espuny-Camacho et al., 2018). These results emphasize the need for NSPC transplants to regionally match the host environment for successful cortical repair.

Cardoso et al. (2018) used transsynaptic tracing to confirm dopaminergic graft integration into appropriate midbrain targets in a rat PD model. The authors grafted ventral midbrain NPSC grafts onto a PD rat model and analyzed the results at different time points. To confirm that the results came from the NPSC graft, the authors injected a monosynaptic tracer into the graft to evaluate the results. At 6 weeks after transplantation, grafted neurons could integrate with host circuitry, forming synaptic connections comparable with endogenous midbrain connectivity. At 24 weeks after transplantation, grafted neurons were shown to extend axonal projections to the appropriate target structures (Cardoso et al., 2018). These results show that this NPSC graft has the capability to integrate into the ventral midbrain host circuitry and potentially achieve functional recovery. The use of transsynaptic tracing methods allows researchers to better study the connectivity between host and graft neurons. Future directions in this field include determining whether synaptic connectivity between graft and host neurons is as cell-type specific as the host neuronal environment and if the connectivity is responsible for the functional efficacy of NSPC therapy (Adler et al., 2020).

Beyond regional identity, the source of transplanted cells itself is an important determinant of outcome. Embryonic stem cell- or fetal tissue-derived cells offer robust differentiation potential but raise ethical concerns, while induced pluripotent stem cells enable autologous transplantation and reduce immunogenicity but raise tumorigenicity concerns. Mesenchymal stem cells, though less prone to neuronal differentiation, have been widely studied for their paracrine and trophic effects. Hence, cell source is a key translational variable that must be considered in the development of human therapies.

**Emergence of new factors defining graft success:** Recent analyses have begun to evaluate how factors such as biological sex, phenotypic identity, and the development of grafts affect outcomes following transplantation. Some of these outcomes include graft maturation and survival, graft differentiation into different neuronal populations, graft axon extension, functional sensory and motor circuit integration into the host neurological environment, and the potential recovery of neurological function. Until recently, the role of biological sex in NSPC grafting was not frequently considered. Pitonak et al. (2022) sought to understand how the biology of transplanted cells influences outcomes in mice SCI models after transplantation. To answer their question, they transplanted sex-matched, sex-mismatched, or mixed donor mouse spinal cord NSPCs into SCI sites of adult female and male mice. They found that male NSPC grafts transplanted into spinal cord lesion sites of female host animals caused an immune response characterized by hypervascularization and cytotoxic T-cell infiltration into grafts (Pitonak et al., 2022). This study was the first to demonstrate that in SCI, as in clinical organ transplantation, the biological sex of the donor can significantly influence outcomes.

In parallel, Liu et al. (2022) applied engineering strategies in traumatic brain injury, using 3D-printed scaffolds seeded with mesenchymal stem cells to improve biocompatibility and connectivity, resulting in cognitive recovery in rats. They found that the biocompatibility allowed for increased neuronal differentiation and synaptic connections to be restored. In another study by Kim et al. (2022), the authors created human embryonic stem cell-derived cerebral organoids to replace the damaged portion of the brain after a mild traumatic brain injury. After implanting the eight-week-old organoids, the authors found increased neuronal differentiation, angiogenesis, and synapse formation between the graft and host circuitry. Together, these results led to the reconstruction of the damaged cortex and improvement in cognitive function (Kim et al., 2022). While the authors from these papers used different methods to enhance biocompatibility between the graft and host neural environment, it subsequently led to improved synapse formation and connectivity amongst the graft itself and the graft with the host neuronal circuitry, and improved neuronal function.

Increasing attention has also been given to vascularization and supporting epithelial populations within grafts. Adequate vascular supply is essential for graft survival, and strategies such as co-transplantation of vascular progenitors or engineered vascularized constructs have been shown to enhance oxygenation, reduce necrosis, and improve functional integration (Li et al., 2024). Incorporating non-neuronal supporting cells represents a critical step toward building grafts that more closely recapitulate native tissue microenvironments.

**Targeted engineering and precision approaches:** Aceves et al. (2023) demonstrated that the developmental age of mouse embryos utilized as donors for spinal cord NSPC grafts can affect graft abundances of diverse interneuronal subtypes and, in turn, host axonal regeneration into grafts. They found that ventralized grafts exhibited greater axon outgrowth and enhanced host serotonergic axon regeneration, while dorsalized grafts exhibited enhanced host axon ingrowth and higher thermal sensitivity, suggesting greater synaptic connectivity of grafts with host sensory systems. These results differed because of neuronal phenotype of the graft. This study demonstrated the importance of how neuronal phenotype of the graft can affect synaptic and functional outcomes. Zholudeva et al. (2024) had a similar thought by realizing the importance of spinal V2a interneurons towards recovery post-SCI, but harvesting and evaluating their therapeutic potential has been difficult to achieve. In their experiment, the authors optically engineered cervical V2a spinal interneurons from human induced pluripotent stem cells and transplanted them into a rat model of the cervical contusion injury. They found that optogenetic activation of transplanted V2a spinal interneurons caused functional synaptic connectivity to the injured host circuits via functional innervation of transplanted cells with host neurons, resulting in improved diaphragm activity assessed by electromyography. Additionally, the *in vivo* environment resulted in the maturation of spinal interneurons that mediate the formation of neuronal relays and differentiation of glial progenitors involved in repairing the host environment. The authors achieved functional improvement by choosing a neuronal phenotype known to play a key role in plasticity and therapy-driven recovery post-SCI. This showed that the driving activity of specific graft neuronal phenotypes can drive recovery.

In a recent study, NSPC grafts expressing the chimeric protein CPTX, a synthetic excitatory synaptic organizer composed of cerebellin-1 and neuronal pentraxin-1, facilitated a significant increase in synapse formation, resulting in significant improvement in locomotion and spinal cord conduction in a rat model of SCI (Saijo et al., 2024). CPTX was also shown to facilitate this improvement in functional synaptic connections through retrograde monosynaptic tracing, indicating that the matured graft was extending to integrate with the host environment neuronal tracts. As a result, the authors concluded that the increased synaptic connectivity between the transplanted neurons and the host environment caused an improvement in locomotion and spinal cord conduction using the Basso, Beattie and Bresnahan locomotor rating scale, testing hindlimb locomotion, and evaluating gait using a treadmill. Without understanding the mechanisms of how synaptic connectivity is achieved or what neuronal circuits should be targeted towards achieving recovery, achieving therapeutic options for all patients, no matter the severity of their disease or injury, will be incredibly difficult. Wang et al. (2024) transplanted induced neural stem cell-derived dopaminergic precursor cells into substantia nigra of PD mice. The cells also included DREADDS to improve synaptic connectivity. After evaluating various mobility tests and electrophysiology, the authors found that the transplanted PD mice established synaptic connections from the transplant with downstream host neurons, and DREADD-dependent activation significantly improved behavioral results. Together, these recent analyses illustrate the “precision” era of NSPC transplantation, where grafts are tailored by phenotype and engineered to optimize connectivity.

**Conclusion:** Throughout this Perspectives article, we have shown how synaptic connectivity is a key factor contributing to functional recovery in various neurological diseases and trauma models. Translation to clinical application in spinal cord injury and other diseases will require not only refining strategies to promote graft-host synaptogenesis, but also overcoming barriers imposed by the injury environment. The immune response and chronic lesion microenvironment strongly influence graft outcomes, with inflammation, adaptive immune rejection, and the glial scar limiting survival and integration. Emerging approaches, including immunomodulation, immune-evasive graft engineering, and supportive biomaterial scaffolds, offer promising avenues to mitigate these challenges and enhance connectivity. In parallel, the geometry of the host lesion constrains graft distribution and integration, and engineered constructs such as 3D-printed scaffolds or hydrogels are being explored to match graft architecture to host anatomy, thereby improving circuit restoration.

Ultimately, the efficacy of NSPC transplantation depends on a convergence of graft-intrinsic and extrinsic factors. Neuronal phenotype, donor cell source, vascularization, immune compatibility, and host lesion geometry each shape the extent of integration and functional benefit. Moving forward into the future, identifying which synaptic connections are most critical for restoring diverse neurological functions will be critical to make grafts more therapeutically effective for disease conditions. A comprehensive understanding of these interdependent variables will accelerate the translation of NSPC-based therapies into clinical outcomes for individuals with SCI and neurological disorders.

*This work was supported by the National Institutes of Health (R01NS116404 to JND) and Mission Connect, a program of TIRR Foundation (#021-101 to JND).*
